# Fracture Resistance of Provisional Crowns: A Finite Element Analysis of a Semi-Permanent Resin—A Pilot Study

**DOI:** 10.3390/dj13040137

**Published:** 2025-03-24

**Authors:** Nadine Kamel, Pascale Habre

**Affiliations:** Department of Prosthodontics, Faculty of Dental Medicine, Saint Joseph University of Beirut, Beirut 1107 2180, Lebanon

**Keywords:** dentistry, prosthodontics, dental prosthesis, temporary, finite element analysis, fractures, stress, computer-aided design, composite resins, poly methyl methacrylate, bruxism

## Abstract

**Background/Objectives**: Fracture resistance is crucial for provisional crowns, especially under high-stress conditions like bruxism. While semi-permanent materials such as Luxacrown are designed for durability, their performance under extreme occlusal forces remains uncertain. This study uses finite element analysis (FEA) to evaluate the fracture resistance of five common provisional crown materials. **Methods**: A standardized digital model of a maxillary first molar was developed with uniform crown thickness. Twenty models were created to assess Unifast Trad (self-curing PMMA), Luxatemp Star (bis-acryl composite), Luxacrown (semi-permanent bis-acryl), Protemp 4 (nanofilled bis-acryl), and Telio CAD (CAD/CAM PMMA). FEA simulations evaluated vertical (250 N), lateral (225 N), diagonal (400 N), and bruxism-level (800 N) forces. Stress-to-strength ratios (SSR) and Von Mises stress distributions were analyzed to evaluate material performance and failure risk. **Results**: Telio CAD exhibited the highest fracture resistance, maintaining SSR values below 100% across scenarios. Luxacrown and Protemp 4 performed adequately under moderate loads but showed increased stress concentrations under bruxism-level forces. Luxatemp Star followed a similar trend, whereas Unifast Trad demonstrated the lowest resistance, accumulating significant stress in all conditions. **Conclusions**: Material selection is key to provisional crown fracture resistance. Telio CAD showed the highest durability, while Luxacrown and Protemp 4 performed well under moderate loads but struggled under extreme forces, raising concerns about semi-permanent materials. Luxatemp Star showed similar trends, and Unifast Trad, the weakest, is best for short-term use.

## 1. Introduction

Provisional dental crowns play a critical role in restorative dentistry by protecting prepared teeth, maintaining esthetics, and preserving functional occlusion before the placement of definitive restorations [1]. In specific clinical scenarios, such as crown lengthening, periodontal therapy, or full-mouth rehabilitation, long-term provisional crowns are necessary to support soft tissue healing, occlusal stabilization, and functional evaluation before the final prosthetic placement [2]. However, one of the primary concerns with these restorations is their mechanical durability, particularly under high-stress conditions such as bruxism, where excessive occlusal forces can lead to premature material failure [3].

Bruxism, a parafunctional habit characterized by involuntary teeth grinding or clenching, has a global prevalence of 22.22%, affecting 21% of individuals during sleep and 23% while awake [4]. The occlusal forces generated in bruxism can range between 600 and 800 N, significantly exceeding normal masticatory forces of 150–250 N. These excessive forces impose compressive, tensile, and shear stresses on provisional crowns, increasing the risk of fracture [5,6]. Given these challenges, traditional provisional materials may not always provide adequate longevity, particularly for patients with parafunctional habits. In response, “semi-permanent” or “long-term” provisional restorations have been developed to enhance durability while remaining distinct from definitive restorations [7].

Unlike traditional provisional crowns, which are typically designed to last up to six months, these restorations are intended to endure longer but still serve as interim solutions before the final prosthesis [8]. They are particularly indicated in complex prosthodontic treatments, progressive loading in implant rehabilitation, and cases where definitive treatment is postponed due to financial or clinical reasons [9]. Additionally, they provide a reliable solution for pediatric patients requiring high esthetic appeal and for orthodontic treatments that demand durable materials with good bonding properties to orthodontic adhesives [10].

The mechanical and functional demands of long-term provisional restorations necessitate careful material selection, considering factors such as fracture resistance, esthetic stability, and wear tolerance. Materials for conventional provisional restorations fall into two major categories: acrylic resins, such as polymethyl methacrylate (PMMA), and composite resins, such as bis-acryl resins, which are based on bisphenol A-glycidyl methacrylate (Bis-GMA) [11]. While PMMA resins are widely used due to their affordability and ease of handling, they exhibit low fracture resistance, making them unsuitable for long-term applications [12]. In contrast, bis-acryl composite resins offer improved mechanical properties and esthetics but still have limitations under extreme occlusal loads [13]. Recent advancements, such as CAD/CAM-milled and 3D-printed PMMA materials, have demonstrated superior mechanical performance and stress distribution, making them promising candidates for long-term provisional restorations [2].

Among the recent materials marketed as semi-permanent, Luxacrown (DMG, Hamburg, Germany) has gained attention due to the manufacturer’s claim that it can last up to five years, significantly longer than conventional provisional materials [14]. However, independent clinical studies evaluating Luxacrown’s long-term durability, particularly under high-stress conditions like bruxism, remain limited [15].

Given the limited independent research on Luxacrown’s long-term performance, this study aims to assess how its mechanical properties compare to conventional provisional materials under simulated occlusal forces. This study evaluates the fracture resistance and stress distribution of five commonly used provisional crown materials under simulated occlusal forces. The primary focus is on comparing Luxacrown to Luxatemp (DMG, Hamburg, Germany) and Protemp 4 (3M ESPE, Seefeld, Germany), as they share a similar bis-acryl composition, to assess whether Luxacrown offers enhanced mechanical properties that justify its classification as a semi-permanent material. Additionally, Luxacrown is evaluated against Telio CAD (Ivoclar Vivadent, Schaan, Liechtenstein), a high-strength CAD/CAM-milled material, to determine whether its performance approaches that of more durable provisional restorations. Unifast Trad (GC, Tokyo, Japan), a self-curing PMMA material, serves as a lower reference point to contextualize the relative performance of all tested materials [16]. Using Finite Element Analysis (FEA), this research analyzes stress distribution and fracture resistance patterns to assess differences in mechanical behavior among the tested materials [17].

## 2. Materials and Methods

### 2.1. Materials

This study evaluated five provisional crown materials—Unifast Trad, Luxatemp Star, Luxacrown, Protemp 4, and Telio CAD—selected based on their widespread clinical use, fabrication methods, and mechanical properties. These materials represent three key categories commonly used in temporary restorations: PMMA-based resins, bis-acryl composite resins, and CAD/CAM resins.

Unifast Trad, a PMMA-based resin, was included for its affordability and common use in short-term applications. However, as a conventional self-curing PMMA material, it is expected to exhibit lower mechanical strength compared to the other materials in this study [12,18]. Telio CAD, a CAD/CAM-fabricated PMMA material, was included as a higher-strength alternative within the PMMA category [2,19]. Luxatemp Star and Protemp 4, both widely used bis-acryl composite resins, were selected to assess mechanical performance within this category [20,21,22,23]. Luxacrown, marketed as a semi-permanent bis-acryl composite with an extended lifespan of up to five years, was included to evaluate whether it offers superior mechanical properties compared to other bis-acryl materials [10,16].

While previous studies have examined the fracture resistance of provisional crowns, the long-term performance of Luxacrown under high-stress conditions, such as bruxism, remains insufficiently studied [10,16]. This study employs FEA to evaluate stress distribution and failure risk under simulated occlusal forces. Given its intended durability, Luxacrown was compared directly with Luxatemp Star and Protemp 4 to determine if it provides a mechanical advantage within its material category.

An overview of the composition, working modes, and manufacturer-claimed lifespans of these materials is presented in Table 1.

The mechanical properties of these materials are critical for evaluating their clinical suitability and behavior under stress. Flexural strength determines resistance to bending forces and is particularly relevant in high-stress scenarios such as bruxism [21,24], while the elastic modulus reflects the material’s stiffness and resistance to deformation [19]. Poisson’s ratio describes how a material deforms perpendicularly when subjected to force, influencing stress distribution [25], and density affects handling properties in clinical applications [26]. These key mechanical properties were incorporated into the FEA simulations to assess stress distribution and fracture resistance under varying occlusal forces. A summary of flexural strength, elastic modulus, Poisson’s ratio, and density is presented in Table 2, providing a basis for evaluating different fabrication techniques and clinical applications under simulated occlusal conditions.

### 2.2. Methods

#### 2.2.1. Model Preparation

**Figure 1:**
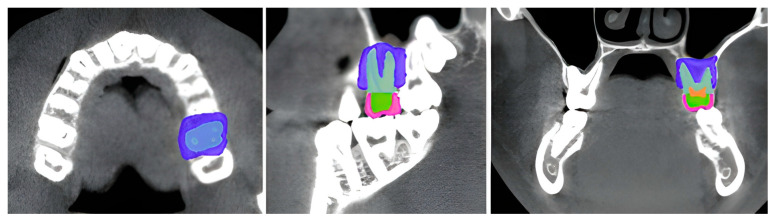
Segmentation of a maxillary first molar from a digital model, showing axial, sagittal, and coronal slices. Different colors represent segmented regions of the tooth and surrounding structures.

A standardized digital model of a maxillary first molar was developed for finite element modeling (FEM) analysis. The model was sourced from the publicly available 3D Slicer sample dataset (PreDentalSurgery.gipl.gz), obtained from the SlicerTestingData repository accessed on 21 March 2025 [31,32]. This dataset is fully anonymized in NRDD format and is pre-approved for research use [33]. The model was processed using 3D Slicer software (version 4.10, Brigham and Women’s Hospital, Harvard Medical School, Boston, MA, USA). Using threshold-based segmentation techniques, individual anatomical structures, including enamel, dentin, pulp, periodontal ligament, and surrounding bone, were isolated and reconstructed into 3D masks. These masks were then merged to generate an anatomically accurate FEM model [34] (Figure 1).

To ensure standardization, clinically relevant dimensions were established, including a crown thickness of 1.5 mm, axial wall thickness of 1 mm, periodontal ligament thickness of 0.25 mm, and cement layer thickness of 0.04 mm, following established guidelines for crown preparation [35,36]. Finally, the material assignment was performed, with each anatomical structure assigned elastic modulus and Poisson’s ratio values based on previously published literature (Table 3).

#### 2.2.2. Finite Element Model (FEM) Preprocessing

**Figure 2:**
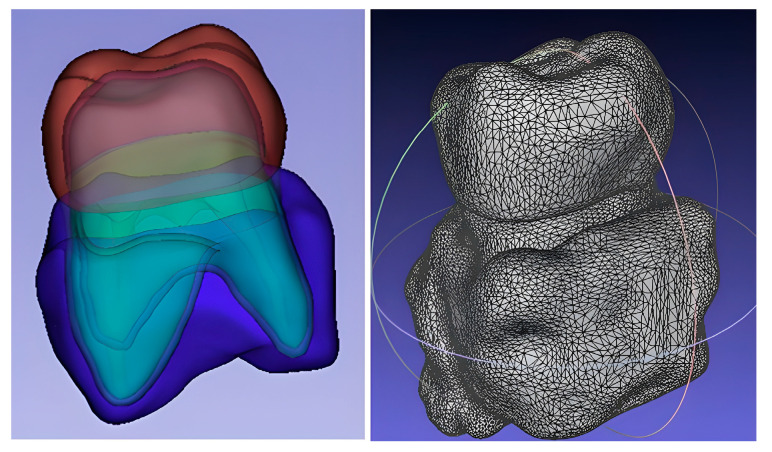
Three-dimensional reconstruction and finite element mesh of the crown model. Different colors represent segmented regions of the tooth and surrounding structures.

Twenty identical FEM models were generated and meshed in Ansys Workbench (version 18.0, Ansys Inc., Canonsburg, PA, USA) using tetrahedral elements to ensure high resolution. Each model consisted of 100,332 elements and 200,640 nodes. FEM preprocessing also included material property assignment, loading conditions, and boundary constraints [41] (Figure 2).

The tested crown materials were modeled as homogeneous, isotropic, and linear-elastic, with their mechanical properties assigned as detailed in Table 2 [10,18,21,22,42].

**Figure 3:**
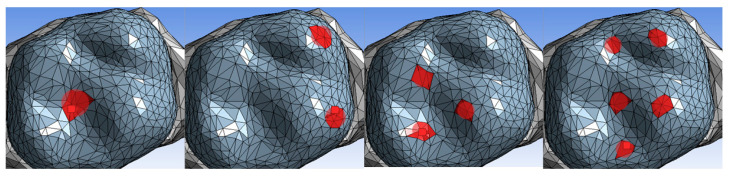
Loading scenarios showing vertical, lateral, diagonal, and bruxism-level forces, with red areas indicating loading points.

To simulate physiological and extreme masticatory forces, four occlusal loading conditions were applied based on FEA, using the Von Mises stress criterion to evaluate stress distribution and identify failure-prone regions (Figure 3). The vertical load of 250 N was selected to represent the average chewing forces [43], while the lateral load of 225 N accounted for forces exerted during lateral mandibular movements, such as grinding [44]. A diagonal load of 400 N was applied to mimic a combined vertical and lateral occlusal force, reflecting a more complex and realistic chewing pattern [45]. Lastly, an extreme bruxism-level load of 800 N was applied to simulate the maximum occlusal forces exerted by individuals with bruxism, as reported in clinical studies of both sleep and awake bruxism [4,46]. The load distribution and stress response of each material were analyzed through FEA simulations to assess their mechanical performance under different occlusal conditions.

For boundary conditions, the base of the bone was fixed to simulate clinical constraints. The periodontal ligament was modeled with an elastic modulus of 0.87 MPa and a thickness of 0.25 mm, allowing for realistic tooth movement [47,48].

#### 2.2.3. Stress Analysis

**Figure 4:**
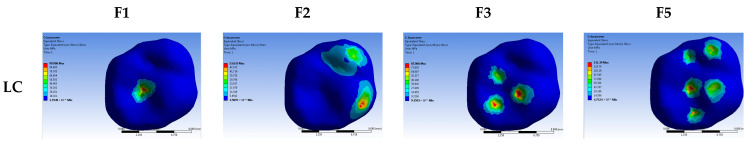
Chromatic stress distribution maps for each material under different load scenarios, highlighting areas of maximum (red) and minimum stress (blue).

Stress distribution was analyzed under simulated occlusal forces using the Von Mises stress criterion, which evaluates a material’s response to complex loading conditions and helps identify regions prone to failure [49]. To quantify the likelihood of material failure, the Stress-to-Strength Ratio (SSR) was calculated for each material under every loading scenario by dividing the maximum stress by the material’s flexural strength (Table 2). An SSR exceeding 100% indicated a high probability of material failure [50]. For visual representation, chromatic stress maps were generated to illustrate stress distribution across the materials. Areas of maximum stress were highlighted in red, while regions experiencing minimal stress appeared in blue [51] (Figure 4).

#### 2.2.4. Data Analysis

The extracted data from the finite FEA simulations included Von Mises stress values and Stress-to-Strength Ratios (SSR) for each material under different occlusal loads. These metrics were used to assess stress distribution and fracture resistance trends across materials. The analysis method focused on evaluating stress concentration areas that may contribute to material failure and estimating the likelihood of fracture under different occlusal loads. Instead of relying on statistical significance, the study interprets results based on mechanical behavior under simulated loading conditions, providing insights into the comparative performance of the tested materials.

## 3. Results

### 3.1. Stress Distribution

Figure 4 illustrates the Von Mises stress distribution under four loading scenarios. TC showed the lowest stress across all conditions, with minimal stress concentrations even under bruxism-level forces (800 N).

LC, PT, and LT performed well under moderate loads but showed increasing stress concentrations near their mechanical limits under bruxism. UF consistently showed the highest stress concentrations, confirming its inferior performance in high-stress conditions.

### 3.2. Stress-to-Strength Ratio (SSR)

The SSR quantifies the percentage of flexural strength used under different loads (Table 4). TC remained below 100% across all conditions, confirming its superior resilience. LC, PT, and LT kept safe SSR values under moderate forces but exceeded 100% under bruxism (LC: 114.25%, PT: 111.10%, LT: 108.66%), indicating susceptibility to failure under extreme conditions.

UF consistently surpassed the fracture threshold, except under vertical loading, with SSR reaching 176.60% under bruxism, confirming its unsuitability for high-stress environments. In contrast, TC remained within the safety limit (SSR: 100.40%), reinforcing its suitability for long-term provisional restorations.

### 3.3. Performance Under Bruxism Loads (800 N)

**Figure 5:**
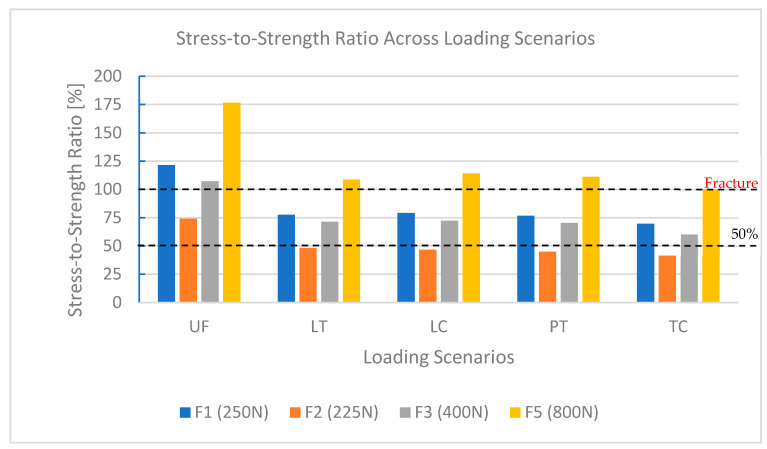
SSR comparison for all materials across simulated forces.

Bruxism-level forces (800 N) further highlighted material performance differences (Figure 5). TC maintained SSR values within safe limits, confirming its suitability for extreme loads. In contrast, LC, LT, and PT exceeded their mechanical limits, which limited their durability under high-stress conditions. UF showed the highest stress levels and SSR values far exceeding 100%, confirming its unsuitability for high-stress applications.

## 4. Discussion

This study assessed the fracture resistance and stress distribution of five provisional crown materials under simulated occlusal forces using FEA. TC showed the highest mechanical stability, reinforcing its suitability for long-term provisional restorations under high loads. LC and PT performed well under moderate forces but failed under bruxism, questioning LC’s semi-permanent classification. LT and UF exhibited the weakest performance, confirming their limitations for short-term applications.

### 4.1. Reliability of FEM Methodology

FEA is a validated tool for assessing dental materials’ mechanical behavior. The Von Mises stress criterion effectively identifies failure-prone regions, supporting its use in occlusal force simulations [52]. However, idealized geometries and standardized forces limit real-world applicability. Patient-specific factors, such as variations in bone density and dynamic occlusion, must be integrated into future studies for greater clinical relevance [53].

Stress values in FEA models are relative and should not be interpreted as absolute failure thresholds. Experimental validation through in vitro testing and thermocycling remains essential for confirming long-term material performance.

### 4.2. Interpretation of Findings

The findings indicate that LC, a bis-acryl composite with 46% glass fillers, exhibited better fracture resistance than LT (44% glass fillers) under moderate loads but failed under extreme bruxism conditions, exceeding 100% SSR. This challenges its classification as a semi-permanent material, as its mechanical limitations contradict manufacturer claims of a five-year lifespan. These findings align with Schwantz et al. (2017), who reported that bis-acryl composites fail under prolonged high-stress conditions [15].

PT, a nanofilled composite resin, outperformed LC in certain scenarios, suggesting that filler content alone does not determine fracture resistance—polymer matrix composition and cross-linking density play key roles.

UF exhibited the weakest performance, accumulating high-stress concentrations and exceeding its failure threshold across most scenarios, consistent with findings on self-curing PMMA’s brittleness [11]. In contrast, TC, a CAD/CAM-milled cross-linked PMMA resin, consistently maintained SSR values below 100%, even under bruxism (800 N), demonstrating superior stress distribution. Its high degree of polymerization, optimized microstructure, and uniform density contribute to its superior stress distribution. These results are consistent with Abad-Coronel et al. (2023), who found CAD/CAM PMMA blocks outperformed bis-acryl composites in fracture resistance [54].

### 4.3. Material Performance and Clinical Implications

Given these findings, TC remains the most suitable material for long-term provisional restorations, particularly in high-stress cases such as bruxism. Its ability to withstand extreme occlusal forces makes it the preferred option for patients requiring extended provisional restorations. These findings align with Niem et al. (2019) and Kassis et al. (2021), who highlighted the durability and energy dissipation properties of CAD/CAM materials under thermocycling conditions, reinforcing their reliability for extended use [1,55]. Additionally, emerging research on composite-based CAD/CAM and 3D-printed materials suggests promising alternatives for future clinical applications [56].

LC and PT performed well under moderate loads but exhibited stress concentrations and exceeded the mechanical threshold under bruxism forces, limiting their long-term durability. While LC is marketed as a semi-permanent material, its failure threshold suggests it may only be viable in patients without excessive occlusal forces. PT, with its nanofilled composite structure, also exhibited moderate stress distribution but lacked sufficient fatigue resistance for bruxism cases. These findings align with Dureja et al. (2018), who noted that bis-acryl composites have acceptable flexural strength but reduced long-term fatigue resistance [9]. LT developed high-stress concentrations under extreme occlusal forces, restricting its use to short-term applications. UF exhibited the weakest performance, making it suitable only for temporary restorations where cost and ease of fabrication are the primary considerations [57].

These findings emphasize the need for material selection based on patient-specific factors, including occlusal forces, expected restoration duration, and functional requirements.

### 4.4. Material Longevity and Manufacturer Claims

While LC is marketed as a semi-permanent material with a lifespan of up to five years, its failure under high-stress conditions suggests that its longevity may be case-dependent. Similarly, although TC exhibits superior mechanical properties, it is only recommended for one year of use. Further clinical validation through thermocycling and fatigue testing is required to determine its actual longevity under intraoral conditions [58]. Additionally, future studies should evaluate other critical properties, such as behavior in acidic environments [59] and long-term color stability [60], to provide a more comprehensive understanding of these materials. Given the limitations of conventional bis-acryl and PMMA-based materials, future research should also explore 3D-printed PMMA and alternative CAD/CAM composites to enhance the durability of provisional restorations [6].

### 4.5. Limitations and Future Directions

While FEA provides valuable insights into stress distribution and fracture risk, its reliance on standardized conditions limits clinical applicability [61]. Future studies should incorporate thermomechanical aging, cyclic fatigue testing, and patient-specific anatomical variability to better simulate real-world conditions. Additionally, validating the SSR metric through in vitro testing and clinical trials is essential for establishing its correlation with actual fracture risk.

Beyond mechanical properties, cost-effectiveness, esthetics, and chairside handling should be considered in material selection. Further research on 3D-printed provisional materials is also needed to assess their mechanical performance and long-term viability.

## 5. Conclusions

This study evaluated the mechanical performance of five provisional crown materials under simulated occlusal forces using FEA, indicating that Telio CAD exhibited the highest fracture resistance, making it the most suitable for high-stress conditions such as bruxism. Protemp 4 and Luxacrown performed well under moderate loads but showed susceptibility to failure under extreme forces, raising concerns about Luxacrown’s long-term durability. Luxatemp Star and Unifast Trad were better suited for short-term applications, with Unifast Trad displaying the weakest performance. Given the limitations of FEA, experimental validation through in vitro testing and long-term studies is necessary to confirm these findings for clinical application.

## Figures and Tables

**Table 1:** Summary of materials evaluated, detailing their composition, working modes, and manufacturer-claimed lifespan.

Code	Material	Composition	Working Mode	Manufacturer-Claimed Lifespan
UF	Unifast Trad (GC, Tokyo, Japan)	PMMA-based resin (powder and liquid)	Direct	Temporary to long-term
LT	Luxatemp Star (DMG, Hamburg, Germany)	Bis-acryl composite (44% glass fillers)	Direct	Short-term
LC	Luxacrown (DMG, Hamburg, Germany)	Bis-acryl composite (46% glass fillers)	Direct	Semi-permanent (≤5 years)
PT	Protemp 4 (3M ESPE, Seefeld, Germany)	Nanofilled composite resin	Direct	Long-term
TC	Telio CAD (Ivoclar Vivadent, Liechtenstein, Schaan, Liechtenstein)	Cross-linked PMMA for CAD/CAM	Indirect	Temporary to long-term

**Table 2:** Mechanical properties of the materials used in finite element modeling (FEM) analysis.

Material Code	Flexural Strength (MPa)	Elastic Modulus (MPa)	Poisson’s Ratio	Density (g/cm^3^ at 20 °C)	References
UF	73	2430	0.43	0.90	[27]
LT	119	3800	0.30	1.50	[28]
LC	115	4100	0.30	1.50	[14]
PT	113	3000	0.30	1.30	[29]
TC	130	3200	0.32	1.20	[30]

**Table 3:** Elastic modulus and Poisson’s ratio of anatomical structures in the FEM model.

Structure	Elastic Modulus (MPa)	Poisson’s Ratio	References
Dentin	16,700	0.31	[37]
Bone	14,700	0.30	[37]
Periodontal Ligament	0.87	0.45	[38]
Pulp	0.0055	0.45	[39]
Resin Cement	16,440	0.26	[40]

**Table 4:** Von Mises stress and SSR values for all loading scenarios.

Material Code	Flexural Resistance (MPa)	Von Mises Stress (MPa) F1, 250 N	Percentage Utilization (F1, %)	Von Mises Stress (MPa) F2, 225 N	Percentage Utilization (F2, %)	Von Mises Stress (MPa) F3, 400 N	Percentage Utilization (F3, %)	Von Mises Stress (MPa) F5, 800 N	Percentage Utilization (F5, %)
UF	73	88.595	121.36	53.963	73.92	78.132	107.03	128.92	176.60
LT	113	87.568	77.49	54.392	48.14	80.671	71.39	122.79	108.66
LC	115	90.906	79.05	53.633	46.64	83.066	72.23	131.39	114.25
PT	119	91.296	76.71	53.353	44.83	83.682	70.23	132.21	111.10
TC	130	90.367	69.51	53.665	41.28	78.526	60.40	130.52	100.40

## Data Availability

The data supporting the reported results of this study are available on request from the corresponding author.

## Abbreviations

The following abbreviations are used in this manuscript:

FEA	Finite Element Analysis
FEM	Finite Element Method
PMMA	Polymethyl Methacrylate
CAD	Computer-Aided Design
CAM	Computer-Aided Manufacturing
NRRD	Nearly Raw Raster Data (file format for volumetric imaging)
SSR	Stress-to-Strength Ratio
